# Harnessing polyphenols from pulp industry residues of juvenile eucalyptus wood: potential for adhesive applications

**DOI:** 10.1186/s40643-025-00914-4

**Published:** 2025-07-11

**Authors:** Lucía Xavier, Rodrigo Coniglio, Fabián Bermúdez, Diego Passarella, Leonardo Clavijo

**Affiliations:** 1 Institute of Chemical Engineering, Faculty of Engineering, Universidad de la República, Julio Herrera y Reissig 565, Montevideo, Uruguay; 2 University Centre of Tacuarembó, Universidad de la República, Route 5, Km: 386.5, Tacuarembó, Uruguay

**Keywords:** *Eucalyptus*, Polyphenols, Adhesives, Valorization, Biorefineries

## Abstract

Approximately 1–2% of the wood processed for pulp production is converted into sawdust and pinchips, which are commonly burned for energy in industries that already produce excess power. This material, juvenile wood only containing sapwood, poses challenges for its valorization. This study explores the extraction of polyphenols from *Eucalyptus* wood residues generated by a pulp industry in Uruguay, aiming to evaluate their suitability as partial replacements in phenol-formaldehyde (PF) adhesives. Using solid-liquid extraction with sodium hydroxide and sodium sulfite, the extractions conditions were optimized through an experimental design considering sodium hydroxide and sodium sulfite charges, along with temperature as variables. The extracts were characterized based on phenolic content, condensed tannins content, antioxidant activity, and Stiasny number, to assess the adhesive potential. Adhesives formulated with 10%, 20%, and 30% PF resin substitution demonstrated a progressive reduction in tensile shear strength, with the 10% substitution achieving acceptable strength. While higher substitution levels compromised adhesive performance, the results suggest that formulation adjustments may improve performance at moderate substitution ratios. Furthermore, the high antioxidant capacity of the extracts opens up potential for their use in other high-value applications where natural antioxidants are increasingly sought after as safer and more sustainable alternatives to synthetic additives.

**Graphical abstract:**

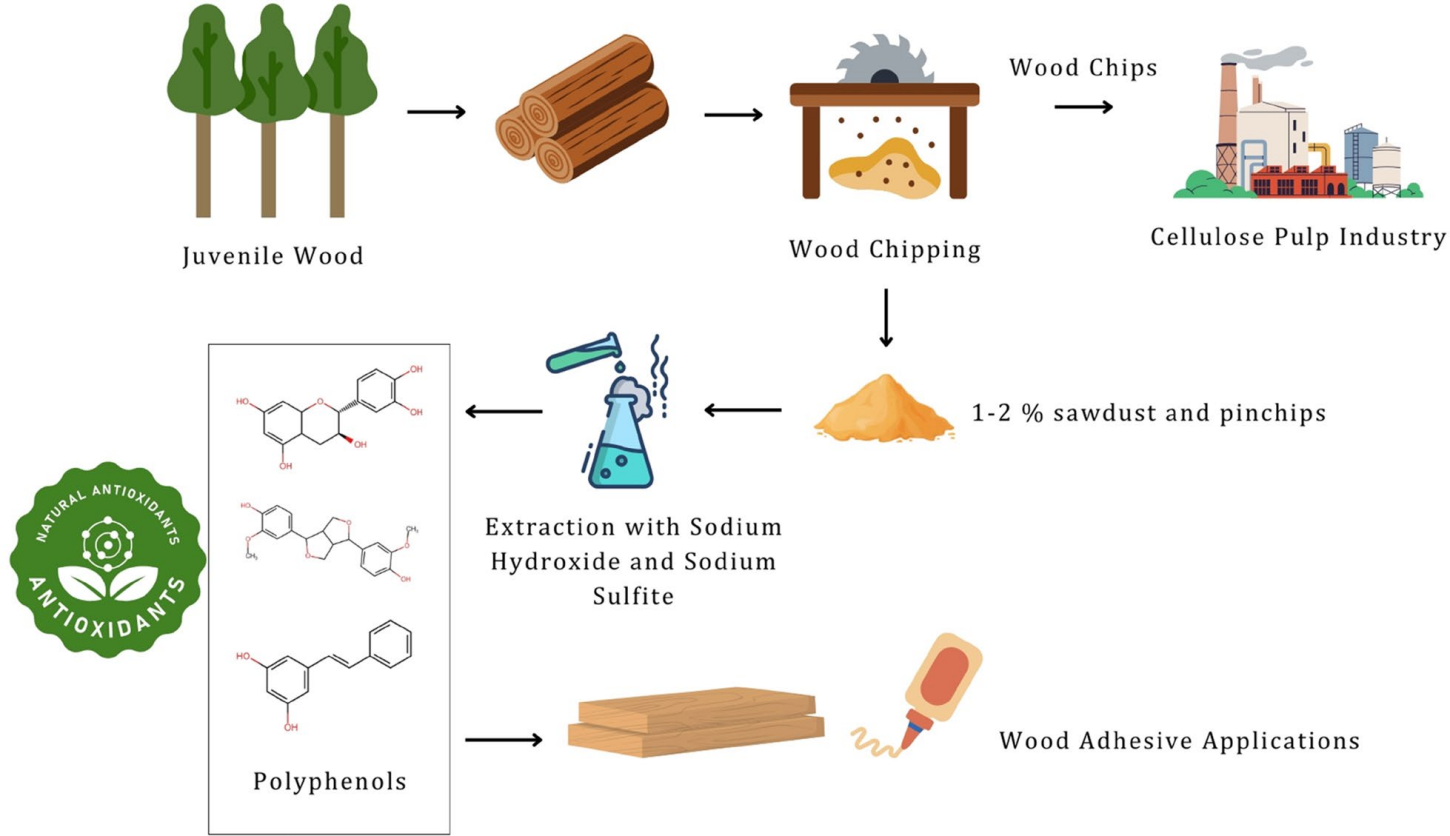

## Introduction

In the last decades, there has been increasing research on the development of new chemicals and fuels from renewable sources, to replace the oil-based production and contribute to the reduction of global warming (Mussatto and Dragone 2016; Rochón et al. 2022). In Uruguay, the pulp industry has been growing consistently since the first mill started to produce in 2007, and the production industry relies on fast-growing wood species, with *Eucalyptus* being the predominant genus utilized across Latin America. Plantations are typically harvested for pulp production at an average age of 10 years, consisting predominantly of juvenile wood (Resquin et al. 2022). Nearly 4.5 million tons of pulp are produced anually (Klein and Luna 2022). However, the raw material is not fully utilized and there is considerable research aimed at developing biorefineries so that the waste and by-products from these industries can also be used to generate high value-added products (Cherubini 2010; Mussatto and Dragone 2016).

During the wood processing phase at the mill, residual materials such as pinchips (particles thicker than 3 mm but still thinner than the accepted 7 mm) and sawdust (fine particles with a thickness below 3 mm) are formed during chipping and usually represent 1–2% of the total wood and cannot be processed (Guigou et al. 2019). These residues (referred as fines) are excluded from the production line due to their lower fiber quality and the physical issues they may cause during pulping. Valorizing these residues to develop value-added products represents a promising opportunity to enhance the sustainability and profitability of the pulp industry (Kumar et al. 2024), given that they are commonly burned in biomass boilers to produce energy. However, pulp mills generate energy in surplus of their operational requirements (Sun et al. 2018; Tuck et al. 2012). Therefore, to improves the revenues, this raw material can be processed to produce biofuels or other chemical feedstocks (Guigou et al. 2019; Cebreiros et al. 2020, 2021; Camesasca et al. 2021; Xavier and Cabrera 2021; Rochón et al. 2022).

The structural components of wood are lignin, cellulose, and hemicelluloses. Furthermore, it has other minor components that can be extracted with solvents and are called extractives (Sjöström 1993; Xavier and Cabrera 2021). Thousands of different extractive species can be found in wood, and their abundance and distribution vary greatly depending on factors such as species, wood age, growth conditions, or even within the same tree depending on the studied section. In this regard, some sections such as bark (Xavier et al. 2021) or knotwood (Coniglio et al. 2023) are richer in extractives than wood. Wood is generally divided into two primary tissues: sapwood and heartwood. Sapwood is the outer, living part of the wood, actively involved in the transport of water and nutrients (sap), whereas heartwood forms as trees mature. By this process known as duraminization, cells in the sapwood die and accumulate extractives which increase resistance to decay and influence properties such as color and durability. Juvenile wood, produced in the early years of growth, has less structural density and a different extractive profile compared to mature wood, often resulting in lower levels of protective compounds found in heartwood (Gierlinger and Wimmer 2004). In commercial pulpwood plantations with rotations of approximately 10 to 12 years, such as those used for our study, only sapwood and juvenile wood are harvested (Zobel and Sprague 2012). For *Eucalyptus* species, extractive content normally increases from around 2% in the first years of growth to around 5% after 10 years (Kasmani et al. 2011). This younger wood lacks duramen and therefore extractives are dominated by compounds associated with rapid growth rather than long-term protective functions (Dünisch et al. 2010). Nevertheless, this is also highly dependent on the species and the growth conditions. Chemetova et al. (2020) reported that even if the condensed tannin content of juvenile Australian blackwood is lower than that of mature wood, the content of phenolic compounds and flavonoids is up to three times higher in juvenile wood.

**Fig. 1:**
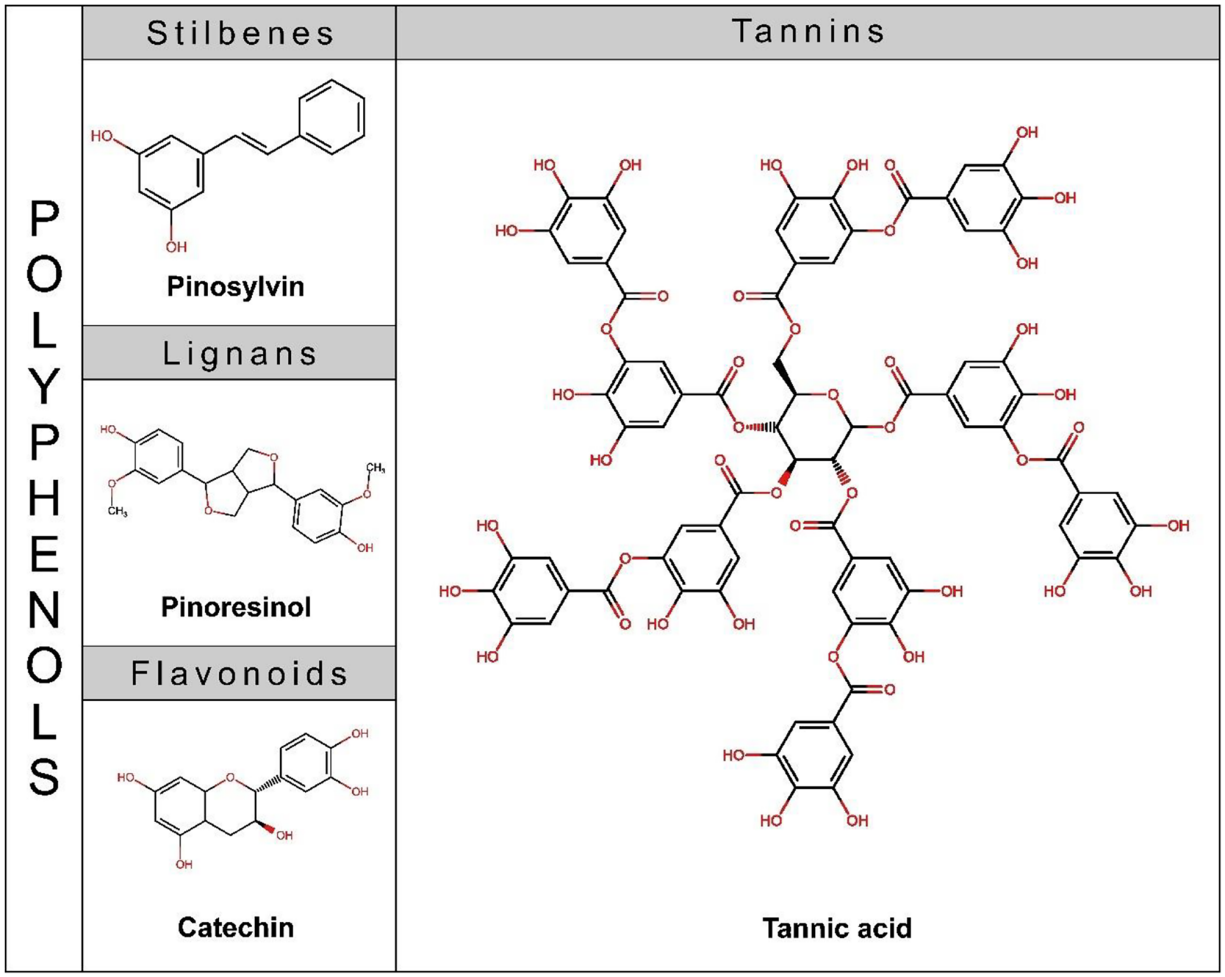
Classification of polyphenols and chemical structure of some representative compounds of each group

There are various classifications for the extractives found in wood, with the term “polyphenols” being widely used in both literature and commercially available products. Although this term encompasses different compounds, it is generally sufficient to consider polyphenols as any aromatic compound containing at least two phenolic units, regardless of the number of hydroxyl substituents (Belščak-Cvitanović et al. 2018). For a long time, this group of compounds was referred to as “tannins.” However, there is now consensus that tannins specifically refer to water-soluble phenolic compounds with molecular weights between 500 Da and 3000 Da that possess the ability to precipitate proteins (Hillis 1987). Therefore, compounds such as stilbenes, lignans, flavonoids, and tannins are commonly grouped within the category of polyphenols (Fig. 1). In some cases, phenolic acids, such as syringic or vanillic acid, as well as other degradation products of lignin, may also be included in this category (Belščak-Cvitanović et al. 2018). Polyphenols are of particular interest and have been significantly studied in the last decades for their antioxidant and antimicrobial properties (Abbas et al. 2017; Bertelli et al. 2021; Manach et al. 2004; Vázquez et al. 2012).

Wood panels such as plywood, Oriented Strand Boards or Laminated Veneer Lumber are mainly manufactured using thermosetting, formaldehyde-based synthetic resins such as phenol-formaldehyde (PF) (Sellers 1985). There are many concerns regarding the emissions of formaldehyde during the curing of the resin as well as from the wood boards (Chupin et al. 2013; Lan et al. 2012; Santos et al. 2018). Furthermore, the need to change the petroleum-based production matrix means that alternatives of renewable origin must be found to replace the phenolic fraction of resins. There has been quite extensive research in this field in recent years (Hemmilä et al. 2017; Norström et al. 2018). Several articles have been published on adhesives based on soy protein (Ghahri et al. 2018; Lorenz et al. 2005; Wescott et al. 2006) and polyphenols extracted from different matrices such as pine bark (Paridah et al. 2002; Santos et al. 2017; Yazaki and Hillis 1977), chestnut (Aires et al. 2016; Vázquez et al. 2010), grape pomace (Lan et al. 2012), *Eucalyptus* bark (Vital et al. 2004), among others.

Most research on bio-based phenolic adhesives has focused on condensed tannins, due to their phenolic structure and ability to react with aldehydes, enabling their use as substitutes for phenol in PF resins (Pizzi 1982; Norström et al. 2018; Dunky 2021; Ma et al. 2021; Zidanes et al. 2023). Their polymerization with formaldehyde enables the production of exterior-grade, weather-resistant adhesives. Alternatives to formaldehyde, such as glyoxal or hexamine, have been developed to reduce emissions while maintaining performance (Pizzi 2016). For instance, tannin–hexamine systems can produce wood panels with low formaldehyde emissions, and autocondensation reactions catalyzed by lignocellulosic surfaces or silica can contribute to bonding in formaldehyde-free systems (Pizzi and Meikleham, 1995; Böhm et al. 2016). The latter also showed that resins cured with more reactive aldehydes (e.g., formaldehyde or glyoxal) show superior network formation, modulus, and stability compared to those relying on autocondensation or low-reactivity aldehydes.

Efforts to simplify processing and valorize underutilized biomass have also led to bark-based adhesives using minimally processed materials. For instance, Santos et al. (2018) produced formaldehyde- and phenol-free adhesives from *Pinus radiata* bark extracts using hexamine as a hardener, achieving exterior-grade plywood panels according to EN 314 (European Committee for Standardization 1993). Likewise, Matsumae et al. (2019) formulated high-performance adhesives using fibrillated radiata pine bark mixed with low amounts of PF resin, suggesting that tannins, hemicelluloses, lignin, and nanocellulose all contribute to bonding.

In contrast to the extensive literature on tannin-based PF adhesives, the adhesive potential of other polyphenol classes—such as flavonoids, lignans, and stilbenes—remains underexplored in PF resin systems. Most studies involving these non-tannin polyphenols focus on alternative adhesive chemistries. For example, Zhang et al. (2024) developed high-performance polyphenol–diisocyanate (PDC) adhesives using natural polyphenol extracts, including epigallocatechin gallate (EGCG), which exhibited excellent bonding across diverse substrates and resistance to solvents and low temperatures. However, their behavior in PF resins has not been systematically studied. Additionally, pinosylvin and pinosylvin monomethyl ether—two typical stilbenes found in pine wood—have been reported to react strongly with formaldehyde in PF and UF resins (Roffael 2016). While stilbene content is low in *Eucalyptus* wood, flavonoids are commonly present and may exhibit similar reactivity to tannins, making them promising candidates for PF adhesive formulations.

In the case of eucalyptus wood fines, the extractive profile is dominated by non-tannin polyphenols due to the juvenile nature of the wood. A previous study using fines from the same pulp mill reported total phenolic contents of up to 49.2 mg GAE/g, while condensed tannins remained between 1.0% and 3.5% (Guigou et al. 2023). The present study evaluates such polyphenol-rich extracts—derived from juvenile *Eucalyptus grandis* wood residues—as partial substitutes in PF resin formulations. This represents a novel contribution, as it explores the performance of undercharacterized non-tannin polyphenols in a traditional PF system, aiming to expand the range of renewable raw materials for adhesive production and enhance the valorization of pulp industry residues within a biorefinery framework.

The most used method to obtain phenolic compounds from natural products is conventional solid-liquid extraction with solvents. For wood, the following are used: water, alkaline solutions with sodium sulfite and/or urea, or combinations of solvents and water (Shirmohammadli et al. 2018; Hoyos-martínez et al., 2019; Faye et al. 2021).

In several studies, sodium sulfite combined with sodium hydroxide has been utilized to enhance polyphenol extraction and improve lignin removal from plant materials. This combination modifies lignin structure, facilitating the release of phenolic compounds from the lignocellulosic matrix and boosting polyphenol yields. Sodium sulfite acts primarily as a delignifying agent, while sodium hydroxide breaks down cell walls, aiding in the solubilization of phenolics. This synergistic approach is particularly effective in accessing and extracting bioactive compounds from lignin-rich biomass (Tomasi et al. 2023; Vázquez, Santos, et al., 2009).

The objective of this work was to study the extraction of polyphenols from *Eucalyptus* sawdust and pin-chips from juvenile sapwood, evaluating the influence of solvent concentration (aqueous solutions of sodium sulfite and sodium hydroxide) and temperature on the yield, phenolic content and Stiasny number. Subsequently, the most promising extraction conditions were selected, and the antioxidant activity of the resulting extract was evaluated to assess its potential as a source of antioxidant compounds of interest for the cosmetic, pharmaceutical, and food industries.

The potential of the selected extract in PF resin-based adhesive formulations was investigated by preparing formulations with 10%, 20%, and 30% PF resin substitutions and comparing with the base adhesive without substitution. The adhesive properties were assessed through measurements of Stiasny number, curing temperature, and tensile strength.

## Materials and methods

### Materials

*Eucalyptus* fines used in this work has been supplied by a cellulose pulp mill located in Fray Bentos, Uruguay, which processes a mixture of *Eucalyptus grandis* and *Eucalyptus dunnii* with some minor presence of other eucalyptus species. The material was dried in an air-flow dryer (De Nardi, Italy) at a temperature of 40 °C to a final humidity of 9.4% (dry basis (d.b).). Then, particles whose size were larger than 500 μm were selected for extraction. The moisture content of the samples was determined by oven drying at 105 °C to constant weight. All determinations were made by triplicate.

### Chemicals

All reagents were American Chemical Society (ACS) quality or higher. Gallic acid, sulphuric acid, sodium carbonate, L-ascorbic acid, Folin–Ciocalteu reagent, iron (III) chloride hexahydrate, hydrochloric acid, acetic acid, sodium acetate 3-hydrate, sodium hydroxide and sodium sulfite were purchased from Merck (Darmstadt, Germany). 1,1-diphenyl-2-picrylhydrazyl (DPPH), vanillin, formaldehyde, methanol, ethanol and HPLC standards were acquired from Sigma (Steinheim, Germany). Trolox (TRE), 2,4,6-tri(2-pyridyl)-S-triazine (TPTZ), catechin hydrate were purchased from Fluka (Steinheim, Germany).

### Raw material characterization

The composition of the fines (extractives, lignin, carbohydrates and ash) was determined following the National Renewable Laboratory (NREL) protocols (Hames et al. 2008; Sluiter et al. 2005, 2012). The extractives were determined by consecutive extraction with water and ethanol. The extractive-free samples were dried and then subjected to acid hydrolysis. Insoluble lignin was measured as the dried weight of the solid fraction of the hydrolysis and soluble lignin was quantified in the liquid fraction with a UV spectrophotometer (Shimadzu, Japan) using a wavelength of 205 nm and an absorptive factor of 15 L/g.cm. Glucose and the sum of xylose, galactose and mannose were determined in the liquid fraction with a HPLC (Shimadzu, Japan) using Bio-Rad Aminex HPX-87 H column and a refractive index detector. Finally, ashes were determined by calcinating the raw material at 575ºC (Sluiter et al. 2008). All components were expressed as g/100 g d.b.

### Solvent extractions

The extractions of phenolic compounds from eucalyptus fines were performed in cylindrical stirred vessels of stainless-steel rotating in a bath with polyethylene glycol, to control temperature (Fibretec Inc., India). The extractions were carried out for one hour, with a solid-liquid ratio of 1/7 (g/mL). The temperature and the concentration of extracting agents were those selected in the experimental design (See Table 1).

In preliminary studies conducted by our group (Guigou et al. 2023), different extraction solvents were evaluated to obtain extracts rich in tannins for adhesive production. That study analyzed various extraction agents (water, alkaline solutions of sodium hydroxide, sodium sulfite, mixtures of both, and mixtures of sodium sulfite and urea) and operating conditions (extractant concentration and temperature) to assess their effect on extraction yield and extract properties. Based on those results, mixtures of sodium sulfite and sodium hydroxide provided the best yields and extract characteristics. Therefore, this solvent system was selected for use in the present study. Additionally, previous reports in the literature have documented the use of similar extractants with successful results (Santos et al. 2017; Vázquez et al. 2010).

Then, the liquid extract was separated from the solid sample by centrifugation at 1000 rpm. Both the liquid extract and the solid were kept at 4 °C until analysed. Phenolic content, Stiasny number, condensable tannin content and antioxidant activity Ferric reducing antioxidant power (FRAP) and DPPH were determined for the liquid extract. Besides, the liquid extract was structurally characterized by gel permeation chromatography (GPC), and Fourier transform infrared spectroscopy (FTIR).

The extraction yield of the solid was determined as the percentage of weight lost by the solid with respect to the weight of the initial material on a dry basis. The results were expressed as g extract/ 100 g material d.b. Finally, the solid was characterized by FTIR spectroscopy. The raw material was characterized by FTIR for comparison.

### Statistical experimental design

A Box-Behnken design (3^3^) experiment was implemented to study the extraction of phenolic compounds from eucalyptus fines fraction. This design implies the performance of 12 experiments and three replicates at the central point. Table 1 shows the three independent variables studied, including their coding and the Table 2 shows the design matrix. The influence of the independent variables (x_1_, Na_2_SO_3_ concentration; x_2_, NaOH concentration, x_3_, temperature) on the dependent variables (Y_j_) was studied: Y_1_: extraction yield (g extract/100 g material d.b.) - Y_2_: total phenol content (g GAE/100 g material d.b.) - Y_3_: Stiasny number. These dependent variables were selected to evaluate the quality of the extracts to be applied in the formulation of adhesives.

**Table 1:** Natural and coded values of the independent variables

Coded level	NaOH charge (w/w%) x_1_	Na_2_SO_3_ charge (w/w%) x_2_	Temperature (º C) x_3_
-1	1	1	80
0	3	3	100
1	5	5	120

The experimental results were analysed by linear regression using the Infostat software (version 2015, InfoStat Group, FCA, Córdoba, Argentina) applying the “backward” elimination method. The response surface mathematical models were fitted to the following polynomial equation:

1 $$\:{Y}_{j}={\beta\:}_{0}+{\sum\:}_{i=1}^{3}{\beta\:}_{i}{x}_{i}^{\text{*}}+{\sum\:}_{i=1}^{2}{\sum\:}_{j=2>i}^{3}{\beta\:}_{ij}{x}_{i}^{\text{*}}{x}_{ij}^{\text{*}}+{\sum\:}_{i=1}^{3}{\beta\:}_{ii}{x}_{ii}^{\text{*}2}$$

where Y_j_ represents the response variable, $\:\beta\:$_0_ is the constant term of the model, *β*_*i*_, $\:\beta\:$_ij,_ $\:\beta\:$_ii_ are the linear, cross-product and quadratic coefficients, respectively. $\:{x}_{i}^{*}$ are the independent variables coded with values between − 1 (lower limit) and + 1 (upper limit), with 0 being the center point of the interval.

The coding was performed according to Eqs. (2)–(4):

2 $$x_i^*=\frac{x_i-x_M}{\Delta x}$$

3 $$x_M=\frac{x_{L l}+x_{L S}}{2}$$

4 $$\Delta x=\frac{x_{L S}-x_{L I}}{2}$$

where x_i_ is the actual value of the independent variable, x_LS_ is the real upper limit, and *X*_*LI*_ is the real lower limit.

The optimization of each dependent variable was carried out by analyzing the three-dimensional response surfaces obtained for the three independent variables, identifying the conditions that maximized the responses based on the observed trends.

### Adhesives formulation

The liquors from the best condition of extraction (see Results and Discussion) were combined and concentrated in a vacuum oven (Cole-Parmer, model G05053-22). The final solid content of the extract was 48.6%. To evaluate the use of extracts in the formulation of adhesives, it was decided to use them not to manufacture the base resin, but as extenders, replacing part of the commercial PF resin. Four formulations of adhesives were prepared: a reference adhesive without further adding of extract (A0) and three formulations with substitutions of 10% (A10), 20% (A20) and 30% (A30) of the solids of the resins with the solids of the extracts.

The base formulation was adapted from literature and the content of water and fillers were adjusted to get a viscosity between 2000 and 4000 cP, suitable to apply the adhesive on the wooden boards with a spatula (Sellers 1985). The Table 2 shows the percentage of each component in the formulations considering only the solid content of each of them. The sodium hydroxide was added as a 50% solution and the PF resin had a solid content of 43.6%. As the behavior of the adhesive, particularly its viscosity, upon substitution of PF resin with polyphenols was initially uncertain, the formulation from Sellers (1985) was used as a reference, and the ratio between walnut shell flour and wheat flour was kept constant. Nevertheless, we acknowledge that these components, as well as water and NaOH content, could be further optimized to improve adhesive performance.

**Table 2:** Formulation of adhesives

Component	% (w/w)			
	Base Formulation (A0)	10% Substitution (A10)	20% Substitution (A20)	30% Substitution (A30)
Walnut flour	12.9	12.9	12.9	12.9
Wheat flour	17.5	17.5	17.5	17.5
PF Resin	66.1	59.5	52.9	46.3
NaOH	3.5	3.5	3.5	3.5
Extract	0	6.6	13.2	19.8
TOTAL	100	100	100	100

To prepare each adhesive, the fillers (walnut and wheat flour) were added to a beaker to be dispersed in water and stirred manually for 5 min, taking care that no lumps were formed. Then, approximately a third of the total resin was added with constant stirring. After 2 min, the sodium hydroxide was added, and the mixture was stirred for 20 min. Finally, the rest of the PF resin and the extract (except for the base formulation) was added and stirred for 2 more minutes.

The viscosity of each formulation was measured in a Brookfield Viscometer (DV2TLV, Brookfield Engineering, USA) and, if needed, more water was added by drop to get viscosity within the accepted range. The Table 3 shows the final viscosity of each adhesive formulation.

**Table 3:** Viscosity of the adhesives prepared

	Base Formulation (A0)	10% Substitution (A10)	20% Substitution (A20)	30% Substitution (A30)
Viscosity (cP)	4302	3878	2712	3114

### Tensile shear strength determination

**Fig. 2:**
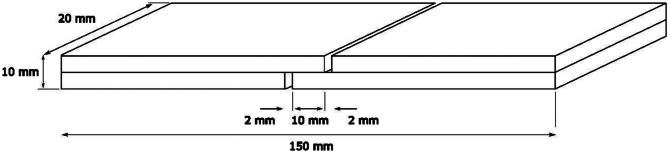
Dimensions of the wooden boards used for the tensile shear strength test showing the cuts done after gluing

Wooden boards were prepared from *Pinus taeda* wood, measuring 150 mm long, 20 mm wide and 5 mm thick (Fig. 2). The tensile shear strength of the joint was measured according to EN 205. For each formulation, 10 test specimens were prepared by gluing two wooden boards with an adhesive load of 230 g/m^2^. Pressing was carried out at 165 °C for 15 min in a hot press (CHY-600DG, Henan Chengyi Laboratory Equipment, China). The pressing temperature was selected after the analysis of the curing temperature of the adhesive formulations. After 5 days two cuts, as shown in Fig. 2,were made to perform the tensile test. Then, all samples were acclimatised for one week at 65% relative humidity and 20ºC. The tensile strength was determined on a Universal Test Machine (Shimadzu, Japan) where the maximum force required to break each specimen was measured, at a rate of traverse of approximately 6 mm/min. Considering a test area of 200 mm^2^, the strength in N/mm^2^ was calculated for each sample.

### Analytical methods

#### Determination of curing temperature of the adhesives

To set the pressing temperature, the curing temperature of each formulation as well as the reference adhesive was determined in a Differential Scanning Calorimeter (DSC 6000, Perkin Elmer, USA). Approximately 10 mg of sample were added to an aluminum capsule, the capsule was sealed and heated from 30ºC to 250ºC with a heating rate of 10ºC/min. The curing temperature was determined as the temperature of the obtained peak for the curing reaction.

#### Phenol content determination

To assess the phenol content, the Folin-Ciocalteu method was employed (Singleton and Rossi 1965). 0.5 mL of extract was added to a test tube, followed by the addition of 2.5 mL of Folin-Ciocalteu reagent (diluted 1:10 v/v) and 2 mL of sodium carbonate solution (75 g/L). The mixture was then incubated at 50 °C for 5 min. Absorbance was recorded at 760 nm using a Shimadzu UV spectrophotometer, with gallic acid as the reference standard for the calibration curve. The total phenolic content was reported as grams of gallic acid equivalents (GAE) per 100 g material d.b.

#### Stiasny number and condensed tannins content determination

The adhesive properties of the extracts were assessed using the Stiasny method. In a round-bottom flask equipped with a condenser, 5 mL of 40% formaldehyde was added to 25 mL of the extract. The mixture was then heated under reflux for 30 min (Yazaki and Hillis 1980). After the reaction, the resulting mixture was filtered through fiberglass filter paper. The solid residue was washed with distilled water and dried at 105 °C until reaching a constant weight. The Stiasny number was calculated as the ratio of the dry mass of the precipitate to the dry mass of the extract.

The results are represented by the following equation:

5 $$\text { Stiasny number }=\frac{P W}{E W} X 100$$

where PW denotes the weight of the precipitate, and EW represents the weight of the extract on a dry basis. The percentage of condensed tannins in the fines is calculated as follows:

6 $$\eqalign{& \>Condensed\>tannins\>(g/100\>{g_{dry\>sawdust}}) \cr & = {{Extraction\>yield\> \times \>Stiasny\>Number} \over {100}} \cr} $$

#### FRAP antioxidant capacity determination

A 0.1 mL sample of the extract was combined with 3 mL of FRAP reagent for analysis. The FRAP reagent was prepared by mixing 25 mL of acetate buffer (300 mmol/L, pH 3.6), 2.5 mL of TPTZ solution (10 mmol/L in 40 mmol/L hydrochloric acid), and 2.5 mL of FeCl₃∙6 H₂O (20 mmol/L). The mixture was incubated for 5 min at 25 °C. Absorbance was then measured at 593 nm using a calibration curve based on ascorbic acid. The antioxidant capacity was reported as nanomoles of ascorbic acid equivalents (AAE) per gram of dry material (Szôllôsi and Szôllôsi Varga 2002).

#### DPPH antioxidant capacity determination

DPPH assays were conducted following the method of Barreira et al. (2008) with slight modifications. A 0.3 mL aliquot of the extract, prepared at varying concentrations, was mixed with 2.7 mL of a DPPH radical solution (6.0 × 10⁻⁵ M in 80% methanol, v/v). The mixture was kept in the dark for 20 min, after which the absorbance was measured at 517 nm. The antioxidant activity was expressed as millimoles of TRE per gram of dry material.

#### FTIR analysis

The samples were measured directly in an FTIR spectrometer (Shimadzu Irafinnity 1 S, Japan) with an ATR accessory, with a resolution of 4 cm^− 1^ and 32 scans per sample, by duplicate.

#### Molecular weight distribution by GPC

To determine the molecular weight distribution, GPC was used. Extracts, as obtained, were filtered through 0.22 mm syringe filters and injected into the HPLC-GPC. A set of PSS MCX columns with porosities of 100 A and 1000 A was used. A pH 12 buffer (NaH_2_PO_4_/NaOH) was used as mobile phase, and a UV detector at 280 nm was employed. Calibration was made with standards of polystyrene sulfonates with molecular weights between 1,000 and 65,000 Da. The analyses were performed in triplicate and the results were averaged.

### Statistical analysis

Determinations were performed in triplicate. The results were expressed as the mean value and its standard deviation. Statistical analyses were performed using the Infostat software (version 2015, InfoStat Group, FCA, Córdoba, Argentina). An analysis of variance (ANOVA) (*p* < 0.05) and the HSD Tukey test (*p* < 0.05) were performed to determine if there was a significant difference between the results obtained. In the design of experiments carried out, an ANOVA was performed to evaluate the significance of the model, where a significance of 90% was set. The determination coefficients (R^2^ and adjusted R^2^) were used to determine the adequacy of the polynomial model.

## Results and discussion

### Characterization of Raw material

The chemical composition of the eucalyputs fines is shown in Table 4. The results are similar to other compositional analysis of the same raw material (Camesasca et al. 2021; Guigou et al. 2019). With regard to the objectives of this work, we can observe that the extractives content is around 5%, an expected value if we take into account the results obtained in other instances working with fines from the same pulp mill. In addition, as already mentioned, the raw material is juvenile wood and containing only sapwood. The result obtained is slightly higher than the 4.3% obtained by Xavier and Cabrera (2021), lower than the 7.1% of Camesasca et al. (2021), and coincides with the 5% of Guigou et al. (2019). Since year to year the proportions of species used by the pulp mill may vary and also considering that growth conditions affect the extractives content, these differences are entirely to be expected. It is also evident that the low percentage of extractives in this type of material becomes a challenge when obtaining useful extracts for the production of adhesives.

**Table 4:** Chemical characterization of the Raw material

Compound	g/100 g material d.b.
Acid soluble lignin	2.9 ± 0.5
Acid insoluble lignin	26.1 ± 0.8
Extractives	5.0 ± 0.8
Ash	0.16 ± 0.02
Glucose	45.1 ± 1.3
Xylose	13.9 ± 0.8
Acetyl groups	4.6 ± 0.7

### Analysis of the influence of extraction conditions through a design of experiments

The influence of Na_2_SO_3_ concentration (x_1_), NaOH concentration (x_2_) and temperature (x_3_) on extraction yield, phenolic content and Stiasny number of extracts obtained with eucalyptus fines was analyzed. Table 5 shows the results of the 15 runs. The objective is to maximize the Stiasny number, together with high values of extraction yield and phenolic content.

The Stiasny number provides valuable information about the reactivity of the extracts toward formaldehyde. Condensed tannins, due to their high content of reactive flavonoid units, can undergo polymerization reactions with formaldehyde, leading to the formation of strong adhesive bonds. A higher Stiasny number indicates greater potential for crosslinking, suggesting that the extract may be more suitable for adhesive formulations (Vázquez et al., 2009; Vieira Pereira et al. 2015; Yazaki et al., 1993).

Using the response surface methodology, the effects of the independent variables selected within the proposed interval were studied. It was observed that the extraction yields varied between 7.61 and 22.17%, the Stiasny number between 7.13 and 42.22% and the phenolic content between 15.01 and 38.66 mg GAE/ g material d.b. Table 6 shows the coefficients of the models according to a second-degree polynomial (Eq. 1), the correlation parameters of the model, their significance, and the standard deviation. Significant models have been found for all the dependent variables studied, with R^2^ values between 0.79 and 0.98. Not all the coefficients of Eq. 1 were significant (*p* > 0.05).

**Table 5:** Experimental design and results of the study variables

EXP	Na_2_SO_3_ charge (w/w%) (x_1_)	NaOH charge (w/w%) (x_2_)	Temperature (ºC) (x_3_)	Extraction yield (g extract/ 100 g material d.b.)	Phenolic content (mg GAE/g material d.b.)	Stiasny number
1	1	1	100	7.61	20.46	42.22
2	1	3	80	9.30	15.01	13.29
3	1	3	120	11.41	27.46	26.99
4	1	5	100	14.24	22.27	18.35
5	3	1	80	11.77	17.65	16.12
6	3	1	120	14.46	22.86	22.84
7	3	5	80	14.84	19.56	7.13
8	3	5	120	20.81	34.89	8.68
9	5	1	100	16.89	31.55	24.76
10	5	3	80	16.93	19.52	7.89
11	5	3	120	20.77	26.97	11.65
12	5	5	100	22.17	38.66	16.20
13	3	3	100	14.82	25.96	21.00
14	3	3	100	15.51	25.58	23.77
15	3	3	100	13.45	26.15	18.89

**Table 6:** Model coefficients and statistical parameters of the experimental design

	Extraction yield (g extract/ 100 g material d.b.)			Phenolic content (mg GAE/g material d.b.)			Stiasny number		
Coefficient	RC	ES	S	RC	ES	S	RC	ES	S
*ß*_*o*_	14.59*	0.288	0.000	27.23*	1.336	0.000	23.60*	1.254	0.000
Linear									
*ß*_*1*_	4.28*	0.269	0.000	3.94*	1.250	0.010	-5.04*	1.173	0.002
*ß*_*2*_	2.67*	0.269	0.000	2.86*	1.250	0.045	-6.90*	1.173	0.000
*ß*_*3*_	1.83*	0.269	0.000	5.06*	1.250	0.002	3.17*	1.173	0.024
Crossproduct									
*ß*_*12*_	-0.34	-	NS	1.33	-	NS	3.83*	1.659	0.046
*ß*_*13*_	0.43	-	NS	-1.25	-	NS	-2.49	-	NS
*ß*_*23*_	0.82*	0.381	0.060	2.53	-	NS	-1.20	-	NS
Quadratic									
*ß*_*11*_	-0.12	-	NS	0.42	-	NS	2.76	-	-
*ß*_*22*_	0.75*	0.394	0.089	1.92	-	NS	1.40	-	-
*ß*_*33*_	0.12	-	NS	-4.08*	1.829	0.043	-9.32*	1.717	0.000
*R* ^*2*^	0.98 0.96 0.76 0			0.79 0.70 3.53 0.002			0.91 0.87 3.31 0		
*R*^*2*^ *adj.*									
*ES*									
*p*									

RC, regression coefficient; ES, error standard; S, significance; NS, not significant (Level of significance: *p* < 0.1)

**Fig. 3:**
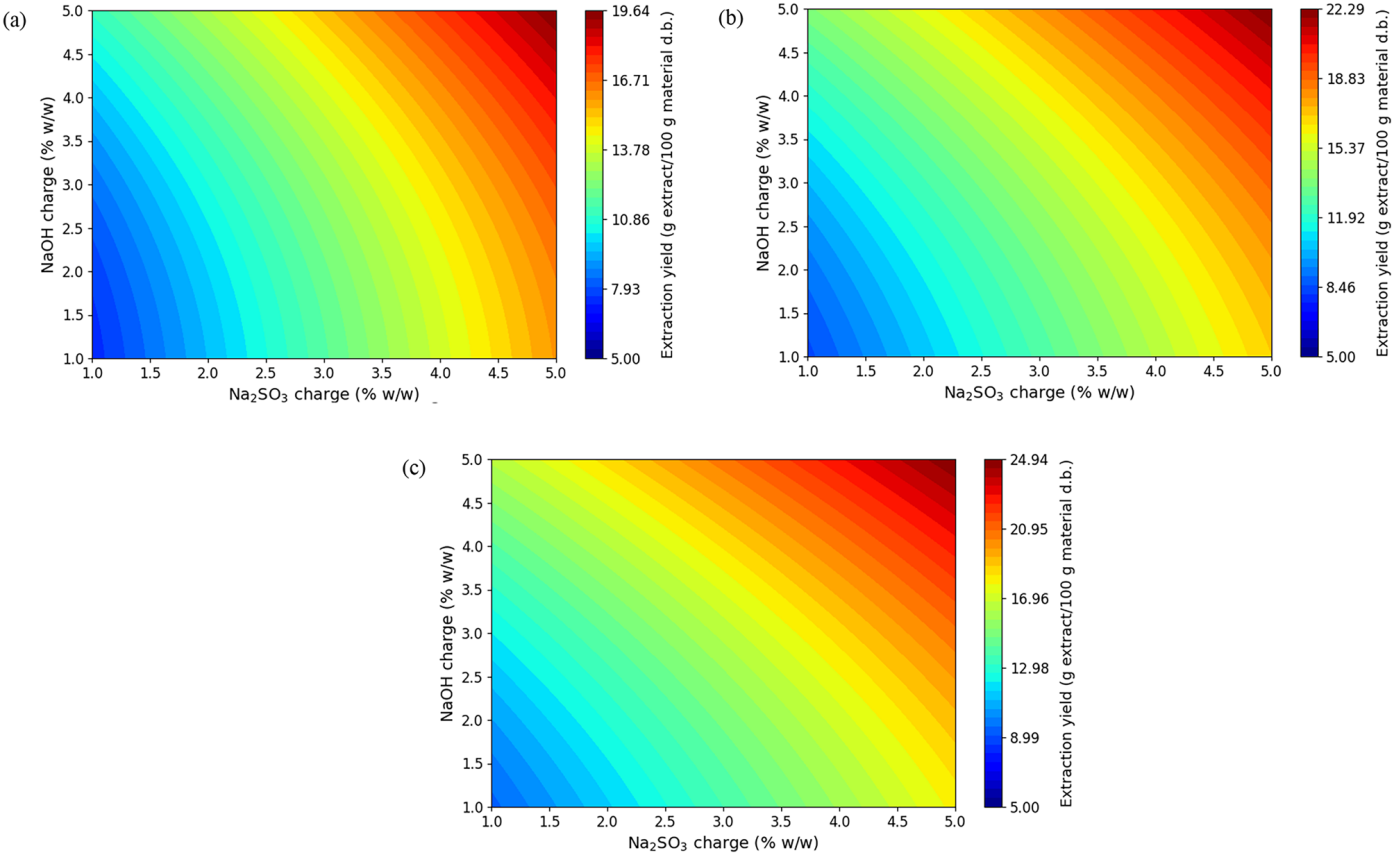
Response surface for extraction yield (Y_1_, %) as a function of Na_2_SO_3_ and NaOH charge (at temperatures 80, 100 and 120ºC)

**Fig. 4:**
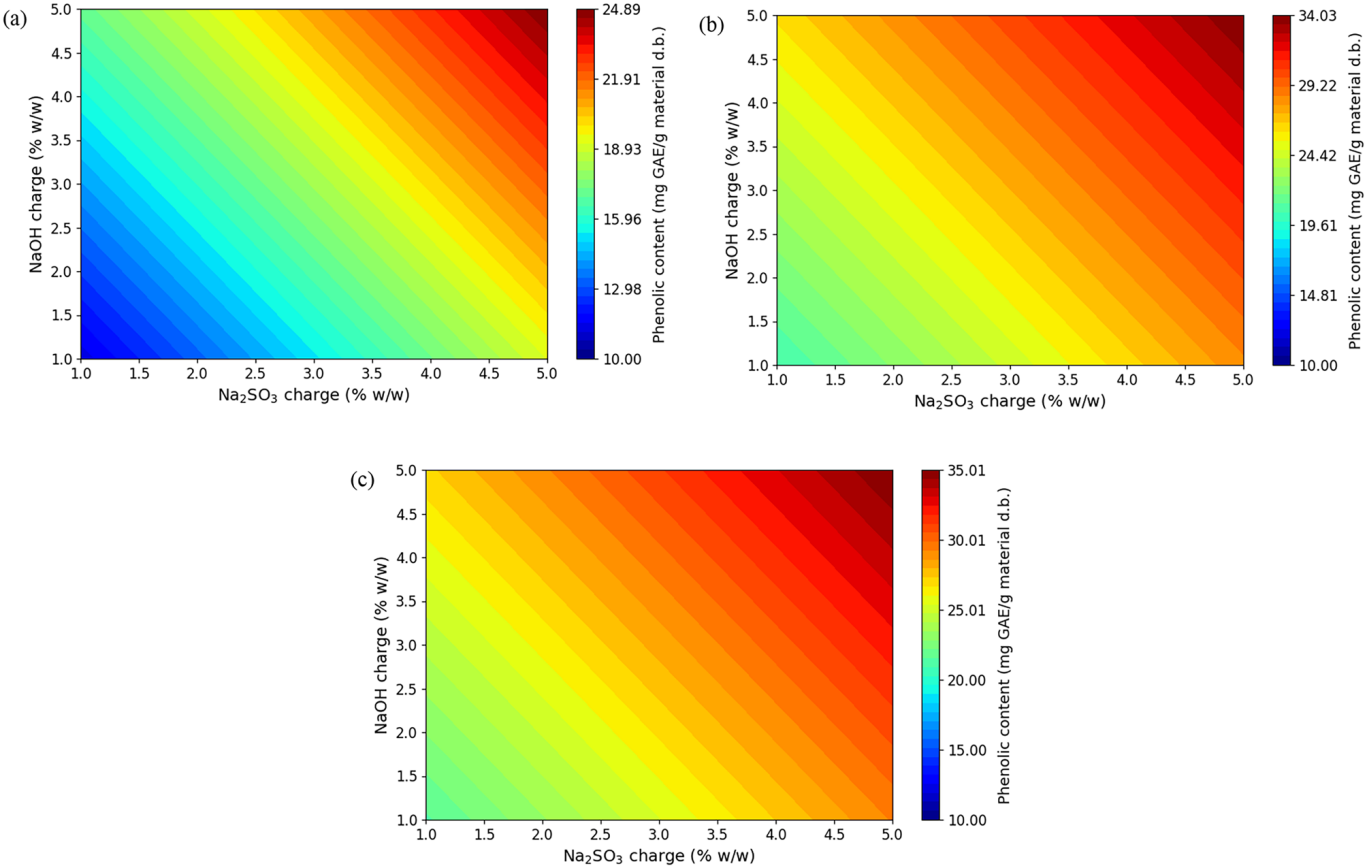
Response surface for phenolic content (Y_2_, mg GAE/ g material d.b.) as a function of Na_2_SO_3_ and NaOH charge (at temperatures 80, 100 and 120ºC)

**Fig. 5:**
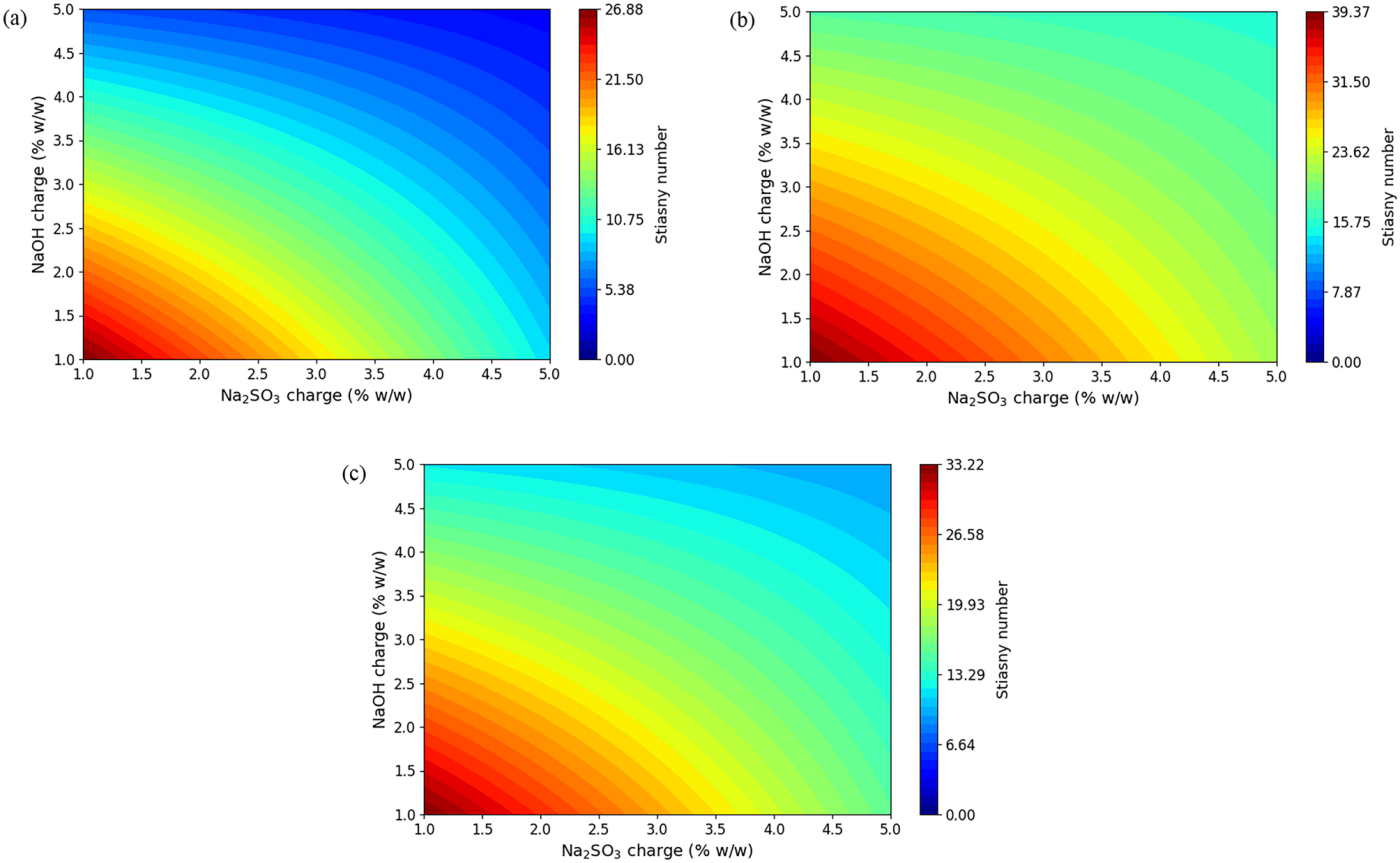
Response surface for the Stiasny number (Y_3_) as a function of Na_2_SO_3_ and NaOH charge (at temperatures 80, 100 and 120ºC)

The response surfaces are shown in Figs. 3, 4 and 5 by contour plots, where the temperature is held constant at each level and the responses are constructed as a function of the charge of Na_2_SO_3_ and NaOH. As shown in Figs. 1 and 2, the extraction yield and the phenol content increase with increasing concentrations of Na_2_SO_3_ and NaOH and also, with increasing temperature. The maximum yield (25.3%) and the maximum phenol content (34.8 mg GAE/ g material d.b.) predicted by the model are obtained for the boundaries of the tested conditions (x_1_, 5%; x_2_, 5%; x_3_, 120 ºC). As expected, the increase in temperature favors the diffusion and solubility of phenolic components.

Santos et al. (2012) studied the extraction of phenolic compounds with mixtures of methanol and water from *Eucalyptus grandis*, *Eucalyptus urograndis* and *Eucalyptus maidenii* bark and obtained lower yields than those found in this work (10.5, 15.2 and 13.2%, respectively). However, the phenolic content obtained in the mentioned work was higher for *Eucalyptus grandis*, *Eucalyptus urograndis* (40.6 and 56.9 mg GAE/ g biomass d.b., respectively) but lower for *Eucalyptus maidenii* bark (26.9 mg GAE/ g biomass d.b.)

The linear effect of temperature, concentration of Na_2_SO_3_ and NaOH positively affected the extraction yield and the phenol content. The interaction between temperature and NaOH concentration was observed only in the extraction yield and had a positive effect on it. Regarding the quadratic effects, the NaOH concentration had a positive effect on the extraction yield and the temperature had a negative effect on the phenolic content.

For the yield the linear effects of the three independent variables were the most significant, followed by the interaction of NaOH with temperature and the quadratic effect of NaOH concentration.

For total phenol content, the linear effect of temperature was found to be the most significant, followed by the linear effect of Na_2_SO_3_ concentration, the quadratic effect of temperature and the linear effect of NaOH concentration.

On the other hand, Fig. 5 shows that the Stiasny number of the extracts increases as the concentrations of Na_2_SO_3_ and NaOH decrease. When the temperature varies, the Stiasny number presents a maximum at 100 ºC. The maximum Stiasny number (39.4) predicted by the model is obtained under the following conditions: (x_1_ = 1%; x_2_ = 1%; x_3_ = 100 ºC). The higher the Stiasny number, the greater the quantity of condensed tannins (Vital et al. 2004). It is not convenient to reduce the Stiasny number, because adhesives with lower viscosity and resistance to the glue line are produced (Vital et al. 2004). The linear effects of Na_2_SO_3_ and NaOH concentration had a negative effect, while the linear effect of temperature had a positive effect on the response. The interaction between the concentration of Na_2_SO_3_ and NaOH caused a positive effect and in the quadratic effects, the temperature exerted a positive effect on the Stiasny number. The most significant effects are the linear effect of NaOH concentration and the quadratic effect of temperature, followed by the linear effects of Na_2_SO_3_ concentration and temperature.

It is concluded that the extraction with the lowest concentration of Na_2_SO_3_ and NaOH leads to the highest values of the Stiasny number, but to a lower extraction yield and phenolic content. The content of dissolved solids and the phenol content are two important extract properties since tannins are part of the extract.

Since the optimal conditions identified varied depending on the response considered, a compromise among the dependent variables studied was necessary. In this case, priority was given to two key parameters: extraction yield and the Stiasny number. A combined response surface was constructed by multiplying, point by point, the values of extraction yield and Stiasny number, with the aim of simultaneously maximizing both responses. This new surface allowed the identification of the combination of factors that maximized the product, which serves as a proportional estimate of the useful condensed tannin content (Eq. 6). The optimal point was determined within the experimental range, selecting the combination of conditions that provided high values for both responses, as these are desirable for obtaining extracts rich in functional tannins.

Therefore, a solution with a high value of the Stiasny number was proposed, but that also allows an acceptable yield and phenolic content. For this reason, it is selected to work at 100 ºC using a NaOH charge of 1% and a Na_2_SO_3_ dose of 3.6%.

To validate the model, the experiments were carried out in the selected working condition, and they were compared with the predicted values of the model. The results are shown in Table 7 where it is observed that the model is adequate to predict the selected dependent variables. Although the Stiasny number does not reach the minimum recommended value (65) for the use of extracts in the formulation of adhesives, it is desired to obtain a sufficiently high value to incorporate the extract in adhesive formulations based on PF resins (Vázquez et al. 2009). The Stiasny number obtained in this work is similar to that obtained by Vázquez et al. (2009) (5.1–37.6) and the one obtained by Mota et al. (2012) (2–41) for *Eucalyptus globulus* bark. The low values obtained from Stiasny number may be due to the hydrolysable character of the tannins (Vázquez et al. 2009).

**Table 7:** Validation of the model in the selected conditions: x_1_: 3.6%, x_2_: 1%, x_3_: 100 ºC

Values	Extraction yield (g extract/ 100 g material d.b.)	Phenolic content (mg GAE/g material d.b.)	Stiasny number
Experimental	13.4 ± 1.1	25.6 ± 0.8	26.5 ± 2.0
Predicted	14.4 ± 0.76	25.6 ± 3.5	27.8 ± 3.3

Predicted and experimental values are presented with a confidence interval (95% confidence level)

Under the selected conditions, the condensed tannin content was determined to be 3.42%, closely aligning with previous results obtained from eucalyptus fines sourced from the same pulp mill (Guigou et al. 2023). In that study, a maximum condensed tannin content of 3.7% was achieved using similar extraction conditions (3.5% Na₂SO₃ and 1% NaOH at 100 °C). As anticipated, these values are considerably lower than those reported for eucalytus bark. Vital et al. (2004) extracted condensed tannins from *Eucalyptus grandis* bark using 4.5% aqueous Na₂SO₃ solutions, reporting a tannin content of 18.47%, while *Eucalyptus pellita* bark yielded 11.38%. This disparity is expected, given the significant differences in extractive composition between bark and wood (Pinto et al. 2013). Tannins play a crucial biological role in protecting the tree against biological threats, such as bacteria, insects, and fungi, which explains their higher concentration in bark, the tree’s primary defensive barrier (Sieniawska and Baj, 2017). Moreover, as this study focuses on juvenile sapwood, the relatively low condensed tannin content is consistent with the characteristics of this material (Kasmani et al. 2011).

### Characterization of the extracts

#### Chemical structure of the extract by FTIR

**Fig. 6:**
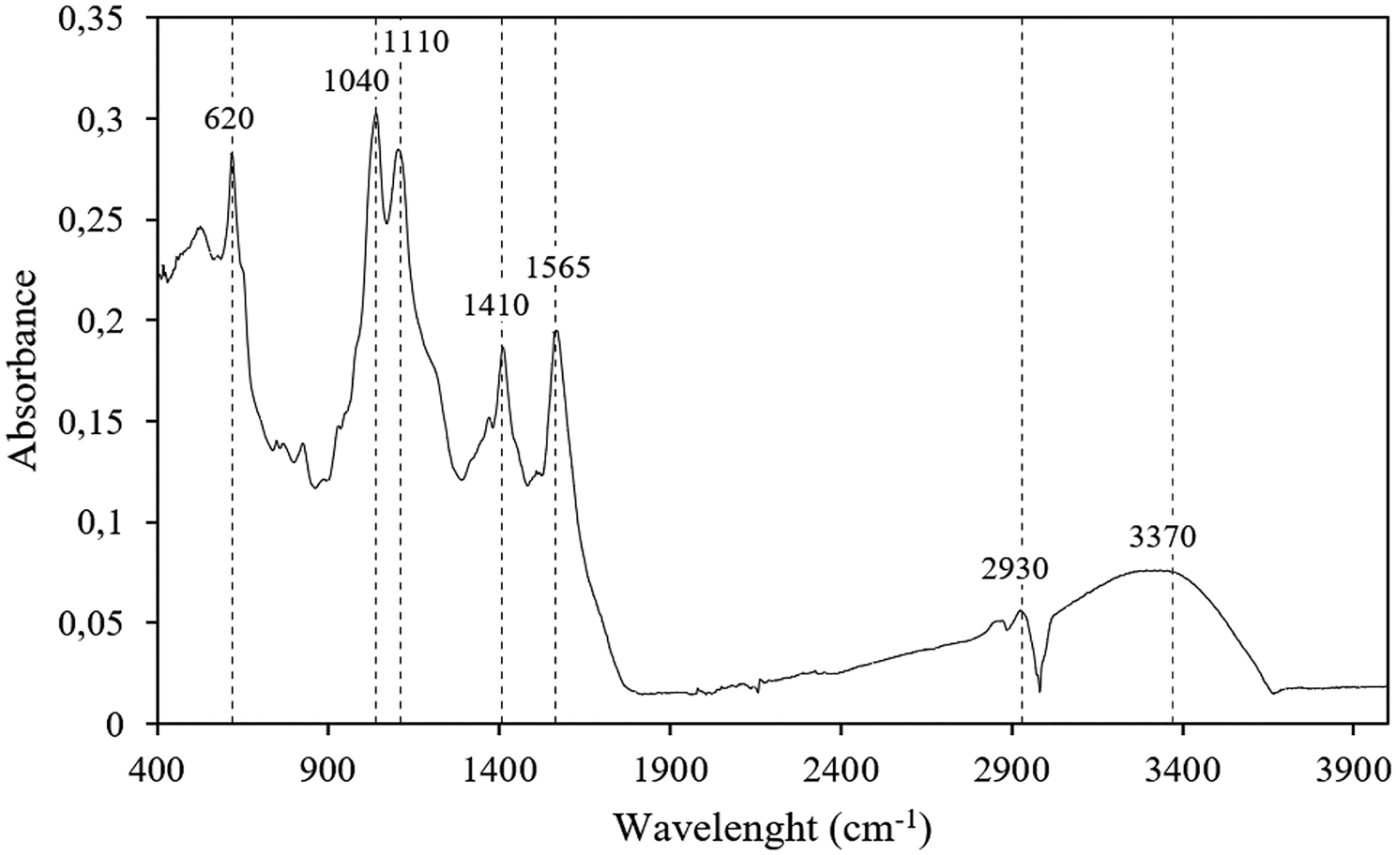
ATR-FTIR spectra of the extract obtained with x_1_ = 3.6%; x_2_ = 1%; x_3_ = 100ºC

The FTIR spectrum of the extract is shown in Fig. 6 under the selected conditions: x_1_ = 3.6%; x_2_ = 1%; x_3_ = 100ºC. Although the FTIR spectrum reports the bonds in all the chemical species of the sample, it is possible to identify some typical bands of functional groups present in the phenolic compounds, which are associated with the vibration frequency of the bonds in the molecules.

A broad band is observed at wavenumbers between 3000 and 3700 cm^− 1^ and corresponds to the vibration due to stretching of OH groups in phenolic and aliphatic structures. The bands in the region between 2930 and 2980 cm^− 1^ correspond to the stretching vibration of the CH_2_ and CH_3_ groups. The bands between 1450 and 1600 cm^− 1^ can be attributed to vibrations of the aromatic skeleton. Near wavelength 1110 cm^− 1^ an intense peak appears that is attributed to the C-H bending vibration in aromatic structures and at 1040 cm^− 1^ another band is shown that corresponds to a C-O stretching vibration (Chupin et al. 2013; Lan et al. 2012). The band at 620 cm⁻¹ can be attributed to aromatic torsion and out-of-plane C–H bending vibrations (Gnassiri Wedaïna et al., 2020).

#### Molecular weight distribution

The molecular weight (M.W.) distribution of the extracted tannins is a critical parameter, as these compounds will be incorporated into the adhesive formulation as extenders by blending them with a preformed PF resin. When both components exhibit similar molecular weights, the curing process of the adhesive tends to be more efficient and effective. This compatibility facilitates the formation of a homogeneous polymer network, enhancing crosslinking density and improving the mechanical properties of the final adhesive. Previous studies have shown that tannins with molecular weights comparable to PF resins actively participate in the curing process, resulting in adhesives with improved performance characteristics (Li et al. 2024; Shnawa et al. 2015). Similarly, low polydispersity coefficients aid adhesive performance when the PF resin is manufactured by replacing part of the phenol with tannins. In our approach, where the tannin is incorporated as an extender, the best performance is achieved when the tannin extract has a polydispersity coefficient similar to that of the commercial PF resin, which is already partially crosslinked.

**Fig. 7:**
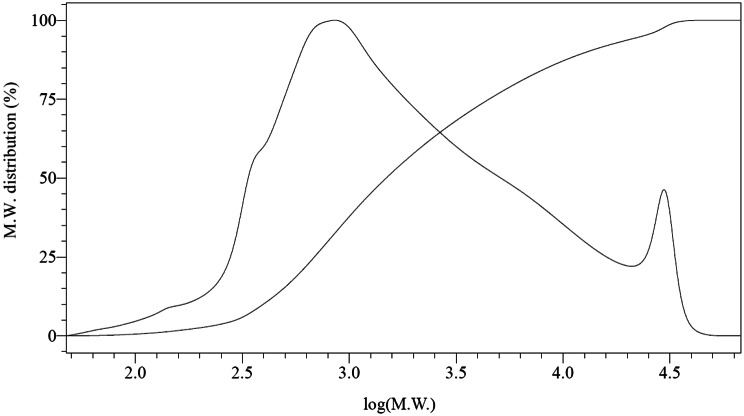
Molecular weight distribution of the extract obtained with x_1_ = 3.6%; x_2_ = 1%; x_3_ = 100 ºC

Figure 7 shows the M.W. distribution of the selected extract. It corresponds to a number average molecular weight Mn = 889 Da, a mass average molecular weight Mw = 4200 Da, and a polydispersity index of PI = 4.7. In the work of (Vázquez et al. 2009) for the extraction of eucalyptus bark with mixtures of NaOH and Na_2_SO_3_ at 70ºC and 90ºC, lower molecular weights and polydispersity coefficients were obtained than those determined in this work (Mn: 400–830 Da, Mw: 1130–1820 Da and PI: 2.1–2.8), although the concentrations of the solutions used were higher. In the work of (Vázquez et al. 1997, 2012) for the extraction of eucalyptus bark with NaOH and Na_2_SO_3_ under conditions like those used in this work, the average molecular weights were also lower (Mn: 500–600 Da). In the work of (Cadahía et al. 1996), wood extracts of *Eucalyptus camaldunensis*, *Eucalyptus globulus* and *Eucalyptus rudis*, obtained with a mixture of methanol-water 80:20 for 24 h at room temperature, were evaluated. In this case, for the three species, higher Mn values were found (1400–1850 Da), lower Mw values (2300–3850 Da), and consequently lower polydispersity coefficients (1.7–2.1).

#### Antioxidant activity

The evaluation of the antioxidant properties of the extracts aims to explore their potential use in other applications of interest in the pharmaceutical, cosmetic, and food industries. Furthermore, many phenolic compounds and tannins are well-known for their antioxidant activity. Measuring this activity provides indirect evidence of their presence in the extract. Several studies in the literature have reported a positive linear correlation between the total phenolic content and antioxidant capacity (Vázquez et al. 2009).

Under the selected conditions, the antioxidant capacity of the extracts was determined, by the FRAP method and the DPPH radical trapping capacity, resulting in a FRAP antioxidant activity of 0.58 ± 0.02 mmol of AAE/g of material d.b. and a DPPH radical inhibition capacity of 0.15 ± 0.01 mmol TRE/g material d.b. The FRAP antioxidant activity was higher than that obtained for *Eucalyptus globulus* bark extracts: Mota et al. (2012) report values of 0.11 mmol AAE/g bark d.b. using mixtures of water with ethanol as solvent and Vázquez et al. (2009) reports values of 0.08 mmol AAE/g bark d.b. with aqueous solutions of Na_2_SO_3_ (Mota et al. 2012; Vázquez et al. 2009). Xavier et al. (2019) reported a FRAP value of 0.18 mmol of AAE/g of material d.b. after performing solid–liquid extraction of phenolic compounds from lignocellulosic forestry by-products using aqueous ethanol solutions, which is lower than the values obtained in the present study. Similarly, Piwowarska et al. (2012), working with the same biomass and using 90% methanol, reported even higher FRAP values, reaching 1.54 mmol of AAE/g d.b.

In the case of DPPH, the values obtained by Fernández-Agullo et al. (2015) for *Eucalyptus globulus* wood were higher than those of this study (0.6 mmol of TRE/ g of wood d.b.) for mixtures of water with methanol. The same authors also tested ethanol–water mixtures, which resulted in even lower values, with a reported DPPH activity of 0.5 mmol of TRE/g d.b. Piwowarska et al. (2012), using lignocellulosic forestry biomass and 90% methanol as the extraction solvent, reported a DPPH value of 0.20 mmol of TRE/g d.b., which is higher than the values obtained in the present study.

### Adhesives

#### Curing temperature of the adhesive formulations

The curing temperatures for each of the formulations of adhesives used are presented in Table 8. In thermosetting adhesives such as PF resins, the curing temperature is a critical parameter, as it defines the point at which the resin transitions from a liquid or fusible state to a crosslinked, infusible network. This process, which occurs primarily through the condensation of hydroxymethyl groups into methylene bridges, is essential to achieving structural integrity, water resistance, and long-term mechanical performance in bonded wood products (Pizzi and Mittal 2003).

**Table 8:** Curing temperature of the formulated adhesives

Adhesive	Base (A0)	10% Substitution (A10)	20% Substitution (A20)	30% Substitution (A30)
Curing temperature (ºC)	149.4	131.1	150.6	141.1

No clear correlation was observed between the curing temperature of the base formulation and its variation upon the addition of the extract. While the formulation with 10% substitution exhibited a reduction in curing temperature, the value increased again at 20% substitution, becoming nearly identical to that of the unmodified PF adhesive. This non-linear trend suggests a curing behavior that differs from typical PF systems modified with phenolic extracts (Kim and Kim 2003). In general, studies involving condensed tannins report a progressive reduction in curing temperature with increasing substitution levels. For example, Hafiz et al. (2020) observed a decrease in the curing temperature of a standard PF resin from 145 °C to 135 °C at 20% tannin substitution, and to 125 °C at 30%, followed by a slight increase to 130 °C at 40%. Similarly, Vázquez et al. (2005), working with phenol–urea–formaldehyde–tannin (PUFT) adhesives, found that curing temperatures consistently dropped from 188 °C for the base resin to 175 °C, 174 °C, 172 °C, and 170 °C with increasing tannin content. Although these studies show that the relationship between substitution level and curing temperature is not always strictly linear, the general trend of decreased curing temperature with tannin addition contrasts with the behavior observed in our system.

This deviation can be attributed to the nature of the extract used in our study, derived from juvenile *Eucalyptus grandis* wood fines. Unlike conventional tannin-rich bark extracts, this material contains relatively low levels of condensed tannins and a broader mix of polyphenols such as flavonoids and lignans, which may have lower reactivity toward formaldehyde. Kollek et al. (1986) showed that the curing kinetics of PF resins are heavily influenced by the availability of hydroxymethyl groups, resin pH, and the degree of precondensation. The introduction of polyphenolic extracts with fewer reactive sites may disrupt the network formation, requiring higher energy input—or higher temperature—to achieve comparable levels of crosslinking.

Moreover, as highlighted by Vázquez et al. (2002), the curing of PF and tannin–PF (TPF) systems follows a complex, multi-step process, in which activation energy varies with the extent of conversion. Their study revealed that even minor changes in formulation or heating rate can shift the onset and rate of curing. While TPF adhesives tend to cure faster due to their favorable structure and viscosity profile, the presence of structurally diverse and less reactive polyphenols in our formulations may reduce the curing rate, explaining the increase in temperature observed at higher substitution levels. The non-linear behavior observed is thus likely a result of competing effects: some components may accelerate initial condensation reactions, while others may hinder complete network development unless compensated by higher curing temperatures.

Therefore, pressing was carried out at 165 °C to ensure that the core of the panels reached a temperature above the measured curing point. This approach was particularly important given the chemical heterogeneity of the polyphenol-rich extracts used, which may contain components of varying reactivity toward formaldehyde. Experimentally determining the curing profile for each adhesive formulation was thus essential to ensure complete polymerization and consistent bonding performance across all substitution levels.

#### Tensile shear strength of the adhesive formulations

Tensile shear strength tests are essential for evaluating the mechanical performance of wood adhesives. This test measures the ability of a bonded joint to withstand combined tensile and shear forces, simulating real-world conditions commonly encountered in both structural and non-structural wood applications. A reduction in tensile shear strength indicates a deterioration in the quality of the adhesive bond. Therefore, minimum threshold values for this parameter are typically established to ensure adequate performance, particularly in applications where mechanical integrity and durability are critical (Dunky 2021; Gedara et al. 2021; Hunt and Dunky 2022).

**Fig. 8:**
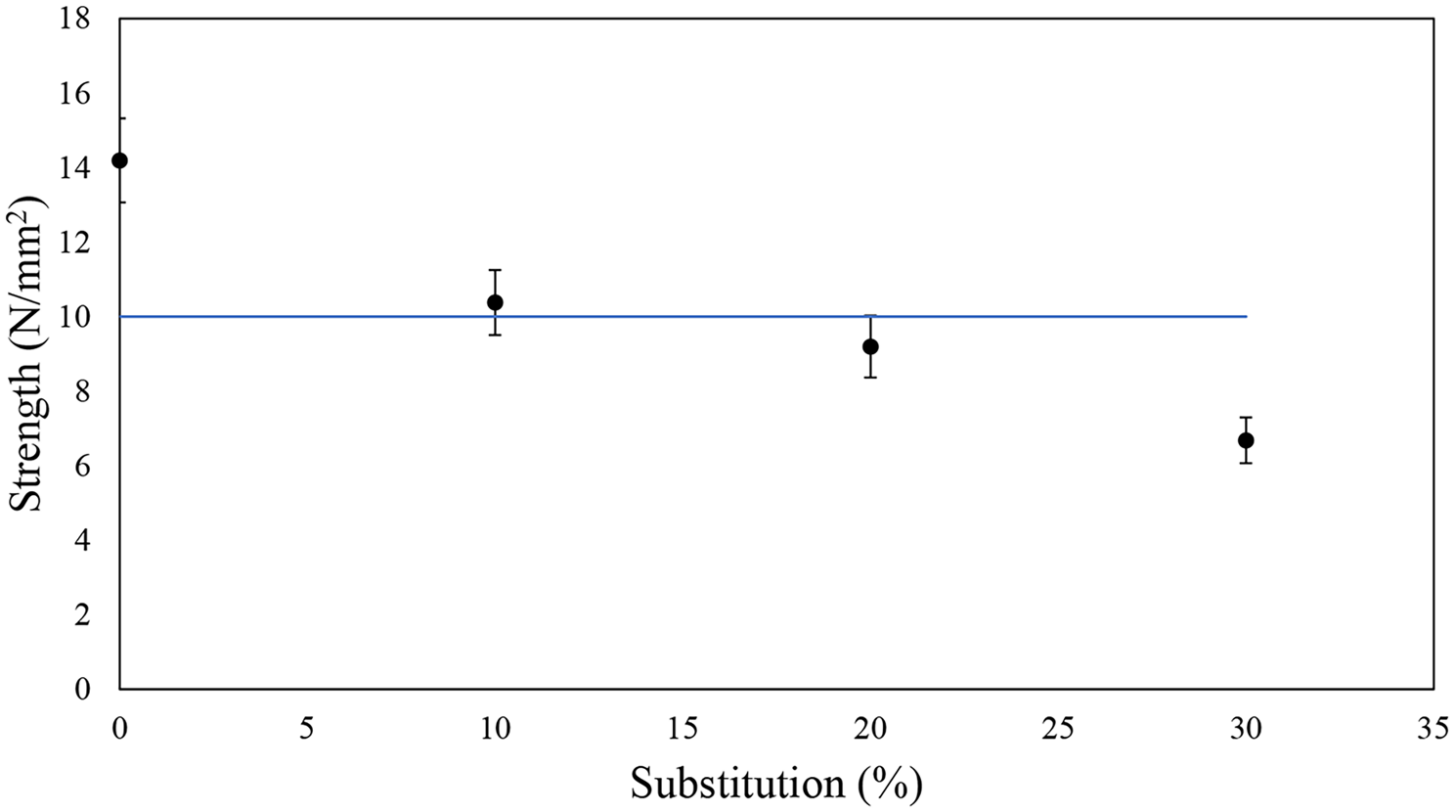
Tensile shear strength of modified adhesives

Figure 8 shows the results of the variation of the tensile shear strength with the percentage of substitution of PF resin by tannins. The strength decreases with the substitution percentage, and for a 30% substitution, values less than half the strength of the base adhesive are obtained. According to the EN 12765:2016 standard, a minimum tensile shear strength of 10 N/mm² must be achieved for samples conditioned for seven days under standard conditions (Saražin et al. 2020; EN 12765, 2016). In the present study, the base adhesive achieved a tensile strength of approximately 14 N/mm², which decreased as polyphenols were incorporated as a substitute. This reduction is expected, as previous studies usually report a decline in strength when PF resin is partially replaced by other bio-based components. For instance, Ugovšek et al. 2010 observed a reduction when incorporating liquefied wood into PF resin, and Özbay and Ayrilmis (2015) found a slight improvement with a 10% bio-oil substitution (12.1 N/mm² compared to 11.1 N/mm² for the base adhesive), followed by a significant reduction to 7.6 N/mm² with a 40% substitution level. The Stiasny number results for the extracts of the present work had already indicated a potential negative impact on adhesive strength, similar to values observed in other studies utilizing extractives in this context. The curing temperature variation also suggested an effect on the resin curing process, so a reduction in tensile strength was anticipated.

For the 10% substitution, tensile strength remained above 10 N/mm², making it acceptable for both structural and non-structural applications. However, at a 20% substitution, the value fell slightly below the acceptable minimum. A 30% substitution resulted in a significant decrease to less than half of the base adhesive’s strength, indicating a pronounced negative effect, making it unsuitable for structural applications.

Tensile strength values are considerably influenced by the adhesive formulation such as viscosity and the use of fillers, as well as gluing conditions. Indeed, Özbay et al. (2015) evaluated different PF resin mixtures with a bio-oil and reported a base adhesive tensile strength just above the minimum threshold (11.05 N/mm²), whereas our base adhesive exceeded 14 N/mm². Optimization of formulations or adjustment of gluing conditions could as well potentially yield acceptable values for the 20% substitution formulation (Saražin et al. 2020).

The low tensile strength obtained with the 30% substitution level suggests that this substitution ratio may be too high, and optimization alone may not achieve acceptable strength levels. However, there is still potential for further development and improvement of application methods.

## Conclusion

This study investigated the extraction of polyphenols from juvenile *Eucalyptus* wood residues, a by-product of the pulp industry, and evaluated their use as a extender in PF adhesives. Despite their relatively low content of condensed tannins, optimized extraction conditions yielded polyphenolic extracts with moderate Stiasny numbers and notable antioxidant activity. These findings confirm that chemically less favorable lignocellulosic materials, such as sapwood from young trees, can still be useful in value-added applications. In adhesive formulations, the incorporation of these extracts as partial PF resin substitutes resulted in a progressive decline in tensile shear strength. However, the 10% substitution level maintained mechanical performance above the standard threshold (10 N/mm²), suitable for non-structural and structural applications. In contrast, the 20% substitution showed a slight drop below the acceptable threshold, while a 30% substitution led to a significant strength reduction, revealing a need for formulation tuning to offset the lower reactivity of non-tannin polyphenols. In the present study, the ratios of walnut shell flour and wheat flour were kept fixed across all formulations. A more exhaustive investigation into the influence of these components on adhesive performance—particularly in terms of mechanical properties and curing behavior—could provide valuable insight for improving performance at higher substitution levels. Additionally, although viscosity was adjusted to fall within the 2000–4000 cP range recommended for manual application, it was not identical across formulations. The 10% formulation, which yielded the best results, exhibited a viscosity of 3878 cP—closer to the base adhesive (4302 cP)—while higher substitution levels deviated further from this value. Therefore, more refined viscosity control—not only by adjusting water content, as done in this study, but also through variation of NaOH dosage and filler proportions—could enhance resin flow, wood penetration, and ultimately tensile strength. These optimization strategies should be explored in future work to unlock the full potential of polyphenol-rich extracts in adhesive systems.

The results also provide new insight into the curing behavior of PF adhesives modified with chemically diverse polyphenols. Unlike typical tannin–PF systems, no clear correlation was observed between substitution level and curing temperature, likely due to the heterogeneous composition of the extracts. This highlights the importance of characterizing not only total phenolic content, but also the chemical nature and reactivity of individual components when designing bio-based adhesives.

Future research should focus on (i) enhancing extract reactivity—through fractionation, chemical modification, or co-additives; (ii) fine-tuning adhesive formulations to improve curing kinetics and mechanical strength at higher substitution levels; and (iii) evaluating long-term durability under environmental stress. Additionally, a techno-economic assessment is needed to determine the viability of integrating this approach into existing pulp mill operations. Beyond adhesives, the strong antioxidant capacity of the extracts also opens up new opportunities for their use in other industrial sectors, such as packaging, coatings, or cosmetics, thereby supporting a broader biorefinery strategy for wood residues.

## Data Availability

The datasets used and/or analysed during the current study are available from the corresponding author on reasonable request.
